# Gas Endeavour: An Innovative Equipment for Estimating Methane Kinetics During *In Vitro* Rumen Fermentation

**DOI:** 10.3390/ani15091331

**Published:** 2025-05-05

**Authors:** Rashid Iqbal, Sheyla Arango, Franco Tagliapietra, Lucia Bailoni

**Affiliations:** 1 Department of Comparative Biomedicine and Food Science (BCA), University of Padova, 35020 Legnaro, PD, Italy; 2 Department of Agronomy, Food, Natural Resources, Animals and Environment, University of Padova, 35020 Legnaro, PD, Italy; franco.tagliapietra@unipd.it

**Keywords:** ruminants, gas emissions, methane kinetics, in vitro techniques

## Abstract

The growing need to reduce methane emissions from ruminants while enhancing feed utilization has driven the development of innovative *in vitro* measurement techniques. This review examines the Gas Endeavour (GES), an automated volumetric apparatus that quantifies both total gas and methane production in real time during rumen fermentation. Utilizing the principles of liquid displacement and buoyancy, the GES integrates a thermostatically controlled water bath, specialized gas flow cells, and an alkaline CO_2_ absorption unit to deliver precise kinetic data on fermentation. Compared to conventional methods—which often rely on manual measurements and post-incubation gas chromatography—the GES provides continuous monitoring and immediate data acquisition, reducing labour and potential errors. This review discusses the system’s design, operational challenges such as controlling headspace pressure and ensuring consistent inoculum preparation, and its applications in both animal nutrition and biomethane potential assessments. The findings suggest that, with further standardization and protocol refinement, the GES could significantly advance research aimed at optimizing feed digestibility and mitigating methane emissions in ruminant production systems.

## Simple Summary

This review discusses a cutting-edge, fully automated device known as the Gas Endeavour System (GES), which measures gas and methane production kinetics during in vitro rumen fermentation. The GES offers real-time, continuous monitoring of fermentation kinetics, making it a valuable tool for assessing feed quality and evaluating methane emissions in ruminant nutrition studies. This review outlines how the system works—including its water bath incubator, flow cells for gas measurement, and CO_2_ absorption unit—and compares its advantages to traditional methods. It also addresses challenges such as the need for standardizing experimental protocols and minimizing errors from headspace pressure and gas condensation. Overall, the GES represents a promising advancement in improving both the efficiency of animal production and efforts to mitigate climate change.

## 1. Introduction

In many regions globally, ruminant livestock production is a vital source of income and food for people. However, it also significantly contributes to greenhouse gas (GHG) emissions, primarily through enteric methane (CH_4_) production. Enteric fermentation, the process by which methanogenic *archaea* in the rumen produce CH_4_ during digestion, accounts for over 90% of CH_4_ emissions from the livestock sector [1]. This represents approximately 40% of the total agricultural GHG emissions, with annual emissions from ruminants estimated between 80 and 95 million tons of CH_4_ [2]. Addressing rumen CH_4_ emissions presents a viable strategy for mitigating climate change. Moreover, CH_4_ emissions represent an energy loss for ruminants, ranging from 2 to 12% of feed energy, depending on diet composition and intake levels [3,4] Consequently, reducing these emissions could enhance the efficiency of animal production systems while benefiting the environment. Precisely, quantifying methane production establishes a foundation for assessing the effectiveness of emission reduction initiatives and facilitates the establishment of achievable targets in this topic [5,6]. This underscores the importance of developing and applying precise methodologies for estimating CH_4_ emissions in ruminant systems.

Over the past few years, some *in vivo* and *in vitro* techniques were developed to measure total gas production (GP), dry matter digestibility, neutral detergent fibre (NDF) degradability, and methane production from different feedstuffs for ruminants. *In vivo* techniques that involve the use of animals have always been considered gold-standard methods and are useful to estimate methane production. Several techniques have been developed to measure methane emissions in ruminants. The respiration chamber (RC) remains a reference method [7,8], where an animal is placed in a chamber, and gas exchange is monitored by comparing the air composition before and after ventilation. Another widely used method is the sulphur hexafluoride (SF6) tracer technique, where a capillary tube collects gas from the animal’s nose while SF6 is released from the rumen, and gas concentration is measured using gas chromatography [7,8] while emission is calculated by the equation used by Johnson et al. [8,9]. The GreenFeed (GF) system trains animals to approach a feeding station that captures their breath emissions [10,11]. Other methods include the sniffer method [4] during milking, ventilated hoods [12] for capturing gases from the head, and a facemask system [13] connected to a gas analyzer. Some of the disadvantages of the *in vivo* techniques are that they are expensive, laborious, and time-consuming. Furthermore, their ethical challenges are significant, primarily due to their reliance on animal testing. Moreover, as the animal is in a stressful environment, it is extremely difficult to apply any feed manipulation experiment. Even though *in vivo* methods are the gold standard in determining methane production, they are unable to explain the kinetics of methane production.

As public interest in animal welfare grows, scientific research increasingly focuses on developing less invasive methods, such as *in vitro* techniques, to accurately measure methane production by ruminants [14]. These methods not only minimize the impact on animals but also provide precise and detailed results. Most *in vitro* techniques are based on the two-stage method developed by Tilley and Terry [15], which simulates rumen conditions such as the temperature, pH, and anaerobic environment. This approach uses rumen inoculum (strained rumen fluid), a buffer to maintain a stable pH, and a nutrient-rich medium to support the growth and activity of ruminal microorganisms.

The end products of fermentation, such as total gas, methane, and volatile fatty acid production, are crucial for understanding how different feedstuffs are fermented in the rumen. However, investigating the kinetics of their production is a critical next step to gaining deeper insights into the metabolic processes driving methane emissions in the rumen.

The primary aim of this review is to examine and discuss the innovative Gas Endeavour (GES) (BPC Instruments, Lund, Sweden) as a modern, automated approach for estimating methane kinetics during *in vitro* rumen fermentation. This paper intends to highlight the system’s operational principles, its applicability in animal nutrition research, and its advantages over traditional *in vitro* and *in vivo* techniques. Moreover, it assesses challenges and considerations related to practical usage, suggesting standardization and refinement to enhance its research utility.

## 2. *In Vitro* Gas Production Techniques (IVGPTs)

These techniques have been widely used to understand the rumen degradability of feed and forages. Rymer has reported a detailed history and methodological considerations of IVGPTs over the time [16]. Along with the increasing interest in GHG emissions, researchers and companies are developing systems to investigate methane emissions using IVGPTs, which is why some traditional systems have been modified to assess methane emissions and methane kinetics [17,18].

### 2.1. Syringe Method (Hohenheim Gas Test)

The pioneering method developed by Menke for measuring *in vitro* gas production employs large, calibrated syringes filled with feed and buffered rumen fluid. In each syringe, 200 mg of feed dry matter is incubated, and GP values are manually recorded after 24 h of incubation at 39 °C [19]. Using these data, Menke formulated equations to estimate the metabolizable energy of feeds. The resulting values have proven to be highly accurate and strongly correlated with those obtained using more recent methods, which are based on dry matter degradability measured both *in vitro* and *in vivo* [20]. To better capture fermentation kinetics, Blümmel and Ørskov [21] modified Menke’s method by incubating the syringes in a thermostatically controlled water bath, ensuring more consistent temperatures throughout gas recording. Blümmel et al. [21] and Makkar et al. [22] further adapted the method by increasing the sample size to 500 mg and doubling the buffer amount. This adjustment minimized temperature drops during gas readings, which is crucial for accurately monitoring GP over time and capturing detailed fermentation kinetics.

### 2.2. In Vitro Methane Measuring Techniques

Several *in vitro* gas measurement techniques [23,24,25] have been developed over time (Table 1), starting from manual gas estimation methods to fully automatic systems [26], to asses the nutritive value of feeds and the use of nutritional strategies to mitigate methane emissions. Most of the GP systems assess methane production at the end of the incubation using techniques like gas chromatography [26]. Pellikaan et al. [18] adopted the method described by Cone et al. [27], modified it to measure methane kinetics by extracting gas from the headspace through gas-tight syringes. Similarly, Ramin and Huhtanen [3] successfully measured methane kinetics from *in vitro* GP measurements by collecting GP at various intervals of incubation, followed by gas chromatography equipped with a thermal conductivity detector (TCD), to then apply a dynamic mechanistic rumen model to predict the methane kinetic parameters. Another fully automatic system developed by Ankom Technology^®^ (Macedon, NY, USA) is the ANKOM^RF^ gas production system. This system allows the maintenance of low pressure inside the bottles (6.9 kPa) [28] as high pressure can affect the end products [29] and the rate and extent of fermentation [20]. It has been used successfully to visualize and analyze GP kinetics, substrate utilization rates, and explore other critical parameters [30,31,32]. However, since this system is also unable to explain the methane kinetics, again, gas chromatography was used to assess the compositional analysis of gas produced during fermentation. Also, this methodology measures the gas pressure, which needs to be converted into moles of gas produced using the ideal gas law equation and then converted it into millilitres of gas produced by using Avogadro’s law. Furthermore, the instrument has other technical drawbacks, such as the leakage of gas and battery failure [33]. Another fully automatic GP system was developed by Muetzel et al. [34], which can measure GP and methane production kinetics in real time without any manual extraction of gas from the headspace of bottles. This system collects and analyzes fermentation gases with a computer-controlled gas chromatograph, rather than allowing them to escape into the atmosphere once a certain pressure threshold is reached, as in previous systems [30].

Recently, Braidot et al. [35] developed a volume-based system that uses an IR gas analyzer to measure the methane concentration from the gases produced in airtight fermentation bottles. Gas volume is tracked by a gas counter, then directed to the analyzer, which accurately detects methane levels under controlled conditions. The headspace within the bottles and tubing ensures consistent gas measurement before analysis.

### 2.3. Limitations of IVGPTs

The availability of rumen fluid to prepare inoculum is one of the limitations of all the *in vitro* approaches, since it is not always readily available due to high management expenses, ethical concerns, the mixing of saliva during the oral stomach tubing technique, or the need for facilities to maintain rumen-fistulated animals [36,37,38]. Similarly, seasonal changes, fluctuations in feed intake and diet composition among individual animals, and differences in harvesting techniques can all result in qualitative discrepancies in rumen fluid collected at different times, even from the same animal. Therefore, a potential approach is to store the rumen fluid in order to minimize the variations and standardize the rumen microbial activity or to use it immediately after rumen extraction. Indeed, storing rumen fluid using 5% of dimethyl sulfoxide and freezing it at −20 °C has resulted in lower GP with lower variability between treatments compared to the use of fresh rumen [31]. These challenges, which affect all aspects of evaluating ruminal fermentation processes, are particularly pronounced when the research objective is to assess methane emissions. The strictly anaerobic microflora, responsible for methanogenesis, is highly sensitive to disturbances caused by the preparation of the microbial inoculum. Any stress experienced by the inoculum leads to a disproportionately greater reduction in methane emissions compared to the overall reduction in GP [39].

Another limitation of *in vitro* techniques that utilize fermentation bottles instead of syringes lies in managing the pressure buildup within the headspace. Specifically, monitoring pressure variations in the headspace is difficult to achieve using manual or semi-automatic methods. Releasing fermentation gases (venting) is crucial to maintaining low pressure inside the bottles and preventing CO_2_ solubilization in the medium [20]. However, this process must also prevent air ingress, which could disrupt anaerobic conditions, and avoid temperature fluctuations, which could compromise the accuracy of gas volume measurements.

Table 1 summarizes the various IVGPTs developed over time for estimating gas and methane production; most of them are based on pressure measurements from fermentation vessels through sensors. However, and specifically for methane production, these systems do not provide kinetic data that describe the extent and rate of digestion from single-sample incubation. They are mostly indirect methods, requiring the analysis of the gas produced at the end of the incubation to determine the proportion of methane, which is then used to calculate the total methane production.

**Table 1:** *In vitro* fermentation parameters and experimental setup that can influence gas production measurements and the composition of fermentation gases (adapted from Yáñez-Ruiz [40]).

Reference	Device	Water-Bath/Air Incubator	Device Volume, mL	Inoculum/Medium Ratio, mL/mL	Buffer Reference	Duration of Incubation, h	Dietary Substrate, Buffered Medium Ratio mg/mL	Gas Venting and Collection	Pressure Control	Gas Measurement and Analysis
Menke et al. [19]	Syringes	Water rotor bath	30	1:2	[19]	24	6.67	Manual, endpoint sampling	Yes, movable glass piston	Manual, no analysis
Theodorou et al. [24]	Bottles	Air incubator	60	1:9	[24]	24–72	2–20	Manual, endpoint sampling	No, pressure increase	Manual, no analysis
Mauricio et al. [36]	Bottles	Air incubator	100	1:9	[24]	n.s.	10.0	Manual, endpoint sampling	No, pressure increase	Manual, no analysis
Pell and Schofield [23]	Bottles, stirred	Air incubator	10	1:4	[41]	n.s.	10.0	Manual endpoint sampling	No, pressure increase	Manual, CH_4_ by GC
Cone et al. [27]	Bottle, shook	Water bath	100	1:2	[42]	48	6.67	Automated, fixed pressure	Yes	Manual,
Davies et al. [43]	Bottles	Air incubator	100	1:9	[24]	n.s.	10.0	Automated, fixed pressure	Yes	n.s., n.s.
Cornou et al. [33]	Bottles	Air incubator	60	1:2	[42]	72	8.33	Automated, fixed pressure	Yes	Manual, no analysis
Muetzel et al. [34]	Bottles	Air incubator	60	1:4	[44,45]	48	10.0	Automated, Automated	Yes	Automated, CH_4_ by GC
Pellikaan et al. [18]	Bottles, shook	Water bath	60	1:2	[42]	72	8.33	Automated, vented pressure	Yes	Manual, CH_4_ by GC
Tagliapietra et al. [20]	Bottles	Air incubator	75	1:2	[42]	144	6.67	Automated, Automated	Yes	Manual, CH4 by GC
Braidot et al. [35]	Bottles, stirred	Water bath	500	1:2	[42]	48	6.67	Automated, Automated	No, volume base	Automated, CH4—infrared

GC: gas chromatograph; n.s.: not specified.

## 3. The Gas Endeavour System

**Figure 1:**
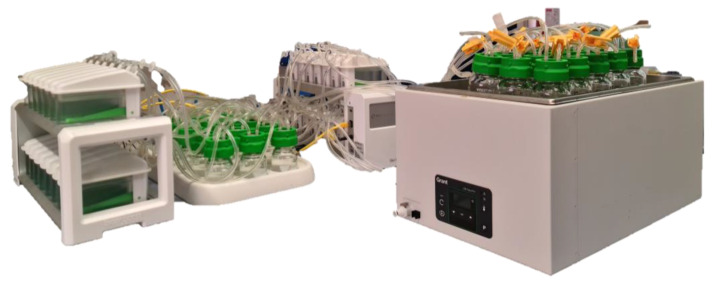
Gas Endeavour System.

The Gas Endeavour (GES), an automatic gas flow measurement system developed by BPC Instruments (Lund, Sweden), is a volumetric gas measurement apparatus capable of detecting low gas volumes (Figure 1) [46]. Initially introduced as the Automatic Methane Potential Test System (AMPTS), it functions as an anaerobic batch fermentation system. The GES operates on the principles of liquid displacement and buoyancy to measure GP. This device allows for the simultaneous analysis of multiple samples within a thermostatically controlled water bath that contains 15–18 digestion bottles continuously shaking at a defined rpm. The water bath, which serves as an incubator, maintains a constant temperature of 39 °C with an automatic heat controller. Samples are weighed directly into the bottles together with the inoculum (rumen fluid) and a buffer solution. The duration of incubation depends on the study’s objectives and typically lasts up to 140 h for feed characterization. The GES estimates not only gas and methane production but also their kinetics. The GES is a real-time instrument designed for measuring the kinetics of both total gas and methane production in the biofuels sector.

In the field of animal nutrition, the GES is still an emerging tool and, compared to other techniques, requires further data validation and the development of standardized protocols to ensure reliability in this context.

### 3.1. Parts and Functionality

**Figure 2:**
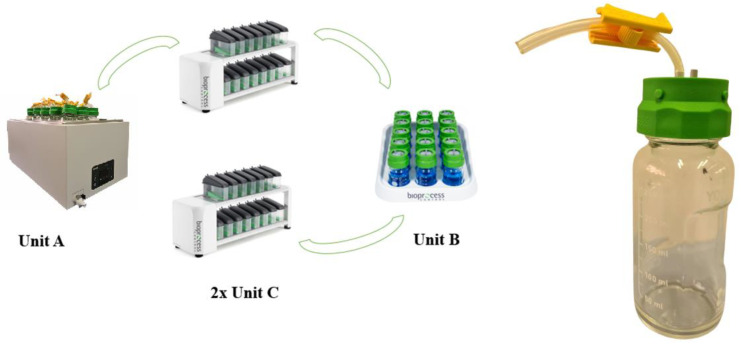
Gas Endeavour parts. Unit A: water bath; Unit B: carbon dioxide absorption unit; Unit C: gas measuring cells and reactor bottle.

Four essential parts (Figure 2) make up the GES: Unit A, Unit B, and two Unit Cs. Then, the whole system has to be connected to a computer to assess to all the functionalities of the system, from settings to results. The equipment described in this review is the one used for animal feed *in vitro* purposes.

Unit A consists of a thermostatic water bath that serves as an incubator, where water temperature and agitation can be set manually before every incubation. Inside of Unit A, 15 bottles or glass reactors with a capacity of 250 mL each contain the sample and other reagents. The bottles are tightly and hermetically closed by a plastic screw cap with two holes or ports (Figure 2). One port is independent; it has just a short Tygon tube with a yellow stopper that allows the opening or closing of the air flow. This is the route from which the liquid reagents can be easily added inside each bottle without opening them and disturbing the anaerobic system. The second hole directly connects each bottle to its own flow cell in Unit C-1 through a Tygon tube. These tubes will transport the gas produced from the bottles of Unit A to the flow cells of Unit C-1.

Unit C is responsible for gas measurement and consists of two units used to measure both total gas (C-1) and methane (C-2). Unit C-1 includes 15–18 flow cell units, each with a measurement resolution of 2 or 9 mL, corresponding to individual bottle reactors. Each cell is equipped with two ports: a gas inlet port that receives gas from the reactor, where every 2 mL of gas displaces liquid, causing a plastic valve to move up and down, which signals a reading. The more frequently this valve moves, the greater the amount of gas being produced. The gas outlet port is connected to Unit B via Tygon tubes, transferring gas that has already been measured by the flow cell. Unit C-2 mirrors the structure of Unit C-1; however, while Unit C-1 is directly connected to Unit A, Unit C-2 is connected to Unit A indirectly via Unit B and Unit C-1 (Figure 2), allowing for methane production measurement.

Unit B serves as the gas absorption unit. It consists of 15 glass bottles, each with a capacity of 100 mL, containing 80 mL of a 3 M NaOH alkaline solution. Each bottle is equipped with a plastic screw cap featuring two ports. One port receives the gas produced by the reactors, which has already passed through the flow cells of Unit C-1. Inside the bottles, carbon dioxide is absorbed by the alkaline solution. The second port of each cap is connected to another flow cell in Unit C-2 via a Tygon tube.

**Figure 3:**
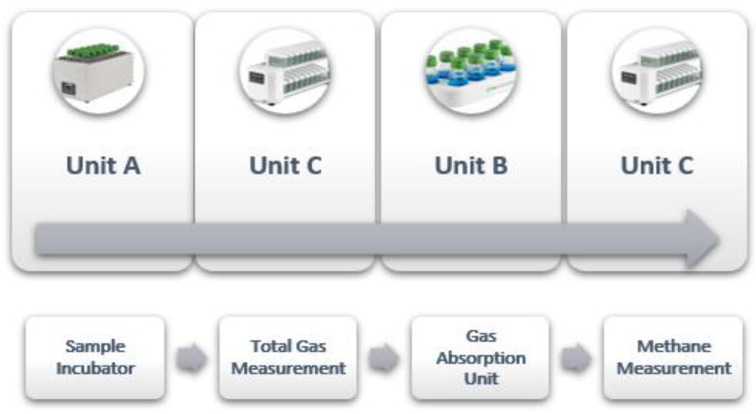
Workflow of the gas in the Gas Endeavour System (adopted from [46]).

Figure 3 explains the workflow of the GES. This scientific arrangement enables the effective investigation and monitoring of the system’s gas dynamics [46]. During and at the end of the incubation, the system provides these parameters: total gas and methane production (both cumulative and at specific intervals, e.g., every 1 min, 5 min, 15 min, 1 h, etc.) and the gas flow rate. Additionally, the system displays a graphical representation of each parameter throughout the whole incubation. All recorded data can be downloaded online as an excel file through a computer connected to the measuring units [47].

### 3.2. Area of Application of the Gas Endeavour System

The GES has been employed across multiple fields to measure gas production (GP), including the assessment of biomethane potential [48,49], biohydrogen potential [50,51], and biomass degradability [52,53] of substrates intended for anaerobic digestion, such as agricultural residues and organic waste. Additionally, the GES is widely used to evaluate the fermentation characteristics of various feedstuffs, including gas production kinetics and methane emissions. Although relatively few studies have explored its application in assessing the quality of feeds and diets for animal feeding and nutrition, promising results have been reported [47,54].

#### 3.2.1. Biomethane Potential

The GES has been successfully used as a regulated laboratory method for determining the biochemical methane potential (BMP) of biodegradable materials automatically, predominantly for measuring the methane coming from the anaerobic fermentation of various organic substances. This technique has been commonly used to measure the methane potential along with the biodegradability status of wastewater and waste biomass from different sources [55,56,57]. Several studies have leveraged Gas Endeavour’s automated data logging and controlled environment capabilities to improve the reliability and reproducibility of BMP assessments. For example, research by Zsuzsanna [48] compared the BMP measured with the GES and the theoretical methane production from using different biodegradable plastics. A non-significant difference was observed between the theoretical and measured biomethane values for one of the products, demonstrating the accuracy of biomethane measurement using the GES. Moreover, the *Gas Endeavour* has been applied across a range of substrates [58], from agricultural residues to food waste, proving versatile in handling the variability in methane production rates typical in BMP tests. Studies, including [56,58,59,60], found that the system’s precision allowed for a detailed kinetic analysis, revealing insights into degradation rates and methane production potential across diverse substrates. This adaptability makes it a preferred choice for researchers aiming to compare substrates under identical conditions.

A comparative analysis between the predecessor of GES, the Automatic Methane Potential Test System (AMPTS II), and the Deutsches Institut für Normung (DIN) method for evaluating total biogas production was performed [61]. Both the AMPTS II and DIN standard methods are batch systems measuring biomethane via gas volume. AMPTS II uses the same principle as GES, while the DIN method uses manual eudiometers (graduated glass tubes) filled with a low-pH buffer to prevent CO_2_ absorption, measuring the total biogas volume by liquid displacement. Methane content is determined periodically via external gas sampling. While both are volume-based batch systems, the key distinction lies in CO_2_ handling—AMPTS II chemically removes CO_2_ for direct methane measurement, the same as GES, whereas the DIN method retains CO_2_ in biogas and measures methane indirectly through manual analysis. Comparative analysis recognizes the GES (AMPTS II) as a new device to analyze biomethane, which is a more automated and rapid method than the DIN. For this test, the GES used 14 bioreactors or bottles with a stirrer incorporated in each cap. The stirring was performed every 30 minutes, the temperature of the water bath was set at 38 °C, and nitrogen was used to flush and purge the Tygon tubes of the instrument. Ten feedstocks were tested: paper sludge, waste jelly, lactose pellets, used cattle bedding, manure scrap, potato sludge, parlour water, fresh straw, cyanobacterial biomass, and hot dog casings. Results showed that eight out of these ten feedstocks were significantly different, but the AMPTS results at 21 days aligned with the DIN results at 28 days. This suggests that AMPTS achieves stable measurements faster, potentially reflecting higher consistency in biogas production, while fresh straw and hot dog casings were not significantly different for total biogas and methane production. The findings revealed notable differences between the systems, warranting further investigation to understand the underlying reasons [61].

Similarly, when the GES was compared with the manual biomethane test. Significantly higher methane production was measured using the GES compared to the BMP. The differences were attributed to the type of method used to measure methane production: manometric (BMP) against volumetric (GES) [62].

#### 3.2.2. Animal Nutrition

After the successful usage of the GES in the biogas sector, researchers are using the GES to measure methane and total gas in animal nutrition to assess the effects of feed additives and forages on methane emissions. Researchers are taking more interest in using this system in animal nutrition, as the system is fully automated and can provide methane production and kinetics in real time [47], while other batch systems, like AnkomRF, require a gas chromatograph to measure methane. In ruminant nutrition research, where understanding the digestibility and methane emissions of feeds is crucial, this instrument plays a key role in evaluating how different feed formulations influence fermentation processes and gas output *in vitro*. For instance, the effect of chopped grass has been investigated on silage, and results showed that the chopping of silage increased the rumen fermentability, resulting in a 9 percent increase in methane production [63]. Researchers from the University of Parma, Italy, used this system for the first time for the purpose of improving ruminant nutrition. They tested multiple feedstuffs to analyze the results of GP from the GES [64]. Similarly, to understand the effect of feed additives on GP kinetics of Bioflavex (Exquim S.A., Barcelona, Spain), a commercial additive made by a mixture of natural flavonoids from which naringina was the main component, taken from *Citrus aurantium* and *Citrus paradisi,* was used as feed additive. The *in vitro* incubation was performed using a TMR for dairy cows producing milk for Parmigiano Reggiano cheese and adding increasing dosages of additives (50, 100, 200, and 400 g/cow/day). The results of total GP at 24 h of *in vitro* incubation using the GES showed that the use of Bioflavex did not affect the total GP in any of the dosages applied. However, the GP between the first and eighth hour of incubation was lower with the use of the additive at the four dosages in comparison to the control diet [65].

The GES has also been utilized to detect and evaluate the ruminal bioavailability and solubility of trace minerals, as they can influence rumen microbial activity, diet fermentation, and the fulfillment of animals’ nutritional requirements. For instance, a study revealed that MnSO_4_, ZnSO_4_, and CuSO_4_ are highly soluble in the rumen, MnO is moderately soluble, and ZnO has low solubility. To enhance the number of replicates, the study employed two GES devices. This bioavailability assessment, based on the trace mineral content, indicated high bioavailability for MnO, MnSO_4_, and CuSO_4_, while ZnO showed poor assimilation by rumen bacteria [66].

Similarly, GES was also used to simulate the large intestine fermentations of different types of dietary fibres and the production of butyrate incubating the substrates in the feces of pigs [67]. Researchers used GES to determine the total GP and took advantage of the kinetics given by the instrument by showing its graphics of cumulative GP profiles. The results showed that the replicates were similar to each other for GP, with a coefficient of variation (CV) of 3.37% at 24 h and 2.65% at 48 h, which may reflect the repeatability of the GES [67].

### 3.3. Challenges and Considerations in the Practical Use of GES

Using the GES in animal nutrition and fermentation studies requires careful attention to experimental details. Given the small volume of gas produced after 24 h of incubation, accurate measurement is essential. While the system’s high sensitivity and automated features provide significant advantages, they also require precise setup and calibration. Below are key challenges and practical considerations to address.

#### 3.3.1. Buffer, Rumen Inoculum, and Feed Substrate Ratio

The volumes of buffered rumen fluid and incubated substrate are directly correlated with GP and methane production kinetics, serving as critical parameters in the GES. The ratios of buffer volume, rumen fluid volume, and substrate amount have been extensively studied and reported in the literature. Menke suggests a rumen-to-buffer ratio of 1:2; for preparing 30 mL of the inoculum; Menke used 0.22 g of diet with 10 mL of rumen liquid and 20 mL of buffer [19]. Van Soest proposes a ratio of 1:4 for preparing 50 mL of inoculum, 0.5 g of diet with 10 mL of rumen fluid and 40 mL of buffer [41]; and Cone adopts Menke’s 1:2 ratio [27]. These ratios substantially influence key factors such as the following: (a) microbial fermentative activity, which determines the efficiency of substrate breakdown; (b) the buffering capacity of the medium, which regulates pH stability and modulates CO_2_ release; and (c) the total gas volume (CO_2_ and CH_4_) produced during fermentation, reflecting the metabolic pathways of the microbial consortia. Despite the broad range of working volumes (medium + inoculum) utilized in biomethane potential studies (ranging from 150 mL to 400 mL, with 200 mL being the most commonly adopted [55,56,58]), the impact of these variations on methane production kinetics in the GES remains insufficiently explored. A systematic investigation into how medium and inoculum volumes influence fermentation dynamics could provide valuable insights.

Recommendation: Standardize the ratio between the buffer, rumen inoculum, and substrate of fermentation in the GES to ensure consistent and reliable results.

#### 3.3.2. Bubbling of CO_2_

The preparation of buffer solutions requires the bubbling of CO_2_, and the duration of this process is a critical factor [19], as it can affect the total GP and kinetics. This effect is due to the absorption of CO_2_ into the buffer solution as carbonic acid and its subsequent release during incubation. While the solubility of CO_2_ in water has been extensively studied [68,69], limited research has been conducted to determine the optimal bubbling duration for CO_2_ in the buffer. Most protocols follow the Menke and Steingass recommendations [19], which suggest the use of resazurin and the bubbling of CO_2_ until colour change, following the continuous flushing of the bottle headspace with CO_2_ during medium preparation to maintain an anaerobic environment. However, significant variability exists among researchers, with some studies reporting CO_2_ bubbling durations of up to 2 h [26,70]. This highlights the lack of standardization in protocols regarding the optimal bubbling time, which could impact the reproducibility and accuracy of the experimental results.

In laboratory routines, the pressure to reduce analysis time can often lead to an underestimation of the critical role of this procedure. It is essential for establishing a strictly anaerobic environment, which is vital for the growth and activity of methanogenic populations. Additionally, this procedure ensures a consistent release of CO_2_ from the buffer, which directly impacts the CO_2_ to CH_4_ ratio in fermentation gases, ultimately influencing the accuracy and reliability of the experimental results.

Recommendation: Standardize the CO_2_ bubbling time during medium preparation to ensure that the medium achieves a complete colour change. This will minimize the risk of underestimating GP and CH_4_ production, particularly during the critical initial hours of incubation.

#### 3.3.3. Effect of Shaking on Gas Production

The agitation or shaking of reactors is an aspect of major concern, as it represents a wide variation across IVGPT methodologies [16]. Mixing plays a crucial role in ensuring the even distribution of microorganisms within the medium, preventing the sedimentation of particulate material, equalizing the temperature distribution inside each bottle, and aiding in the release of trapped gas from bottles. Agitation methods include stirring, shaking, or vigorously agitating. Some authors perform agitation manually or with devices like magnetic stir bars. Practices vary widely: some protocols call for agitation only once, typically at the start of incubation, others do so at fixed intervals, often when measurements are taken, while some procedures omit agitation altogether or fail to report it. In the literature, there is a lack of experimental evidence on the effects of agitation on fermentation processes and the release of fermentation gas. The GES, with its ability to customize times, speed, and the rhythm of agitation, provides a valuable tool for investigating optimal operating conditions and developing guidelines for standardizing *in vitro* methods.

**Recommendation:** The shaking or stirring speed should be controlled to prevent the deposition of dissolved substances or particulates (diet) onto the headspace area or bottle cap. Such deposition may obstruct the gas outlet and leave residues of unfermented diet, potentially leading to inaccurate gas and digestibility measurements.

#### 3.3.4. Effect of Headspace Pressure on Gas Production

In pressure-based GP systems [25,71] there is a potential for underestimating GP as incubation progresses. The increasing pressure from the gas produced within the bottles promotes the solubilization of CO_2_ into the fermentation liquid as carbonic acid, potentially leading to buffer CO_2_ oversaturation [72]. This acidification of the medium selectively inhibits microbial activity and can alter the accuracy of gas volume measurements and impact the interpretation of fermentation kinetics. However, the introduction of gas venting in automatic pressure-based GP systems has addressed this issue [20,30]. Venting the gas accumulated in the bottle headspace is a critical step for ensuring the accurate measurement of GP, as each release carries the risk of introducing measurement errors. In contrast, the GES, a volume-based approach, avoids venting gas into the atmosphere. Instead, it transfers the gas to a measurement unit, maintaining constant pressure in the reactor bottles. Despite these advantages, a detailed study is necessary to evaluate the potential for CO_2_ solubility in both the inoculum and the water present in the GES measurement cells. This is particularly relevant given the solubility of CO_2_ in water, which is 0.231 mmol L⁻^1^ kPa⁻^1^ at 37 °C, as dictated by Henry’s Law [73]. Nevertheless, preliminary findings from our lab (unpublished data) indicate that the reduction in bottle headspace provides more repeatable GP and CH_4_ kinetics in the GES.

Recommendation: Use the smallest possible bottle headspace volume to optimize conditions for accurate GP, specifically for methane measurements with the GES.

#### 3.3.5. Effect of Headspace Pressure on Methane Production

Methane production kinetics can be measured using various procedures, with the most common being as follows: (a) Batch systems without gas release: analysis of the composition of gases accumulated in the headspace of the fermentation bottle. (b) Batch systems with gas release: analysis of both gases accumulated in the headspace and those collected in a dedicated gas bag for released gases. (c) Batch systems with an alkaline trap: the trap removes CO_2_ from the fermentation gas, enabling the measurement of gas volume, which is assumed to consist exclusively of CH_4_ [20,74]. The main limitations of these procedures are as follows: “a”: The increase in headspace pressure enhances CO_2_ solubilization into the medium. This process can lead to an underestimation of GP measurements and an overestimation of CH_4_ proportions (%). ”b”: This method involves dual analysis, which increases the variability of experimental results due to potential air contamination and errors during gas sampling. “c”: This procedure is utilized in the GES and presents the following challenges: (i) potential air contamination (mainly N_2_ and O_2_) in the headspace and tubing cannot be recognized, leading to the overestimation of CH_4_ measurements; (ii) delaying of CH_4_ production measurement compared to GP measurements with both a distortion of their kinetics and an underestimation of the CH_4_ proportion (%).

The last issue has been addressed in the Gas Endeavour system (GES) through the over- and underestimation function, which allows users to input “headspace gas concentration”, “initial flushing headspace concentration”, and “final headspace concentration” [75]. However, the impact of this function on methane kinetics calculations requires further analysis. On the other hand, pressure-based systems like AnkomRF avoid issues of methane over- or underestimation entirely, as these systems do not measure methane kinetics directly.

Recommendation: Remove air from the bottle headspace and the tubes of connection, flushing the system with CO_2_. Minimize the bottle headspace volume to reduce the risk of interference from potential air contamination. Incubate at least 1 g of substrate to minimize the impact of potential air contamination on GP and CH_4_ production and proportions.

#### 3.3.6. Normalization of Gas and Methane Measurement for Temperature, Pressure, and Vapour Interference

According to universal gas law, the volume of gas is directly proportional to temperature and atmospheric pressure, and small changes in these operative conditions can lead to variation in gas measurement, resulting in different GP and methane kinetics. Moreover, temperature influences the production of water vapours; i.e., at a temperature of 39 °C, around 7% of the gas volume is occupied by water vapours [76].

Historical work conducted by Menke to estimate the energy value of feeds using GP measurements was based on data collected at a standardized temperature of 39 °C but without any control on atmospheric pressure. Manual gas measurement systems using bottles cannot standardize the temperature and pressure during gas collection and measurement, as the gas is typically measured at room temperature. In contrast, automated gas measurement systems enable temperature standardization, allowing gases to be measured either at 39 °C or at room temperature, and allow the normalization of atmospheric pressure. In particular, the GES measures gases at room temperature.

In general, according to ISO 10780, gas volumes must always be expressed in “normal conditions”, i.e., at 273.16 K (0 °C) and 101.3 kPa; however, most scientific publications in animal nutrition use data standardized to 39 °C and do not report any information on atmospheric pressure conditions. To solve this problem, gas measurement should be corrected at “normal conditions” for temperature and pressure, also avoiding the effect of vapour production. In the GES, users can measure the gas volume in both conditions, normalized and non-normalized [75], resulting in more accurate gas measurements.

Recommendation: Standardize the laboratory temperature and adjust GP and CH_4_ data to 39 °C to ensure consistency with the majority of the available scientific literature. However, the data provided by the GES at 273.16 K (0 °C) and 101.3 kPa are more accurate gas measurements.

### 3.4. Gas Endeavour Properties and Characteristics: Strengths and Weaknesses

#### 3.4.1. Gas and Methane Measurements

The primary purpose of the GES in animal nutrition is to measure GP, CH_4_ production, and their kinetics during *in vitro* fermentation. This is achieved using sensors and software that continuously monitors and logs data into a computer. Once the experiment begins, no further human intervention is required until the incubation is completed. The equipment offers a sensitivity of 2 mL and a measurement uncertainty declared by the producer of 1% [75], ensuring high accuracy. This accuracy is supported by the already described key design elements, including the gas venting mechanism, controlled bottle environment, and continuous shaking in Unit A.

Unlike other *in vitro* techniques, which require additional analyses such as gas chromatography to determine the methane content at the end of incubation, the GES provides continuous methane production measurements throughout the process. The integration of the system with Unit C allows direct methane quantification, reducing time-consuming and resource-intensive post-incubation analyses.

Another opportunity offered by the GES is the format of its results. While other IVGPTs often report gas production in terms of pressure (psi or kPa), requiring conversion to volume using specific equations, the GES directly provides results in the volume (mL) of gas and methane produced. This eliminates conversion steps, streamlining data interpretation and reducing errors.

#### 3.4.2. Gas and CH_4_ Flow Rate and Kinetics

The rate at which a feed or its chemical components ferment in the rumen is as crucial as the extent of digestion. Feeds with similar overall degradability can ferment at different rates, leading to variations in ruminal dynamics such as rumen bulking capacity, passage rate, and nutrient availability for microbial growth. It has been demonstrated [77] that cumulative gas production after 24 h of incubation does not necessarily correlate with fermentation rates. Therefore, the GP rate is significantly affected by feed intake, and consequently, dairy performances in terms of milk production and quality, as well as methane emissions expressed as daily production (mL/d) or related to milk yield (mL/kg milk) [78].

While many *in vitro* techniques require sophisticated equipment to record GP at different incubation times, the GES measures the GP flow inside each fermentation reactor, monitoring the rate of gas production at any time of the incubation.

Similarly, the *in vitro* CH_4_ production is often evaluated at a single time point, typically at the end of the incubation, leaving limited scientific data on the kinetics of methane formation during fermentation [79]. An advantage of the GES is that the kinetics of fermentation, of gas and methane, can be studied on the same sample, enabling the computation of changes in methane production rates over time. For instance, the GES was used to study the rumen fibre fermentation of soybean and maize silages, evaluating GP kinetics [80]. Similarly, the potential and rate of methane production have been investigated for components of corn stover, including stem bark (SB), stem pith (SP), and leaves (LV) over time [81].

#### 3.4.3. Experimental Design and Results of the Demonstrative Trial

To demonstrate the GES results, a sample trial was conducted using a standard Total Mixed Ration (TMR) formulated to meet nutrient requirements [82]. The TMR consisted of corn silage, mixed meadow hay, alfalfa hay, wheat straw, an energy mix (corn and wheat), a protein mix (soybean and sunflower), wheat germ, and molasses. The detailed chemical composition of the TMR is presented in Table 2.

Rumen fluid was collected from three clinically healthy Italian Simmental cows housed at the “Lucio Toniolo” experimental farm of the University of Padua, Legnaro, Italy. The animals were in mid-lactation with an average of 225 ± 48 days in milk (DIM) and had a parity of two, indicating that they were in their second lactation. Their average body weight was 654 ± 24 kg, and their body condition score (BCS) was 3.41 ± 0.34. The cows produced 27 ± 4 kg of milk per day and consumed approximately 23 ± 4 kg of dry matter daily. All animals were between 35 and 46 months of age and were kept under uniform management, nutritional, and housing conditions throughout the trial period. The study protocol, including animal handling procedures, was approved by the Ethical Committee of the University of Padua (OPBA protocol number 1312041/2022).

**Figure 4:**
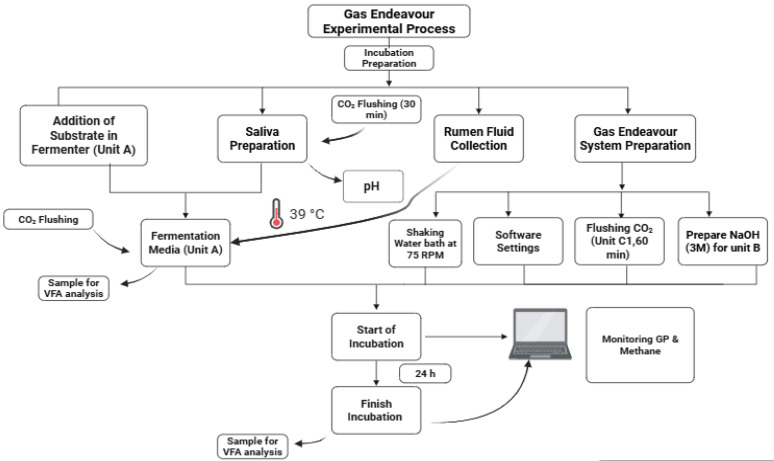
A flowchart of the experimental process displaying all the procedures of the Gas Endeavour.

The experiment included two consecutive *in vitro* incubation trials conducted over two weeks. Each trial consisted of two replicates of the TMR and two blank controls (no feed), resulting in a total of eight fermentation bottles (2 TMR replicates × 2 runs + 2 blanks). The procedure followed the protocol described by Menke and Steingass [42]. Fermentations were conducted in 322 mL bottles, each containing 1.67 ± 0.01 g of ground TMR, 166.67 mL of buffer solution (pre-warmed and pre-flushed with CO_2_), and 83.333 mL of freshly filtered rumen fluid. The bottle headspaces were flushed with CO_2_ and sealed to maintain anaerobic conditions. All setup and sample preparation steps were completed within 40 min of rumen fluid collection. The bottles were incubated at 39 °C, and gas production was measured at defined intervals to assess fermentation kinetics. A visual schematic of the entire procedure is provided in Figure 4.

**Figure 5:**
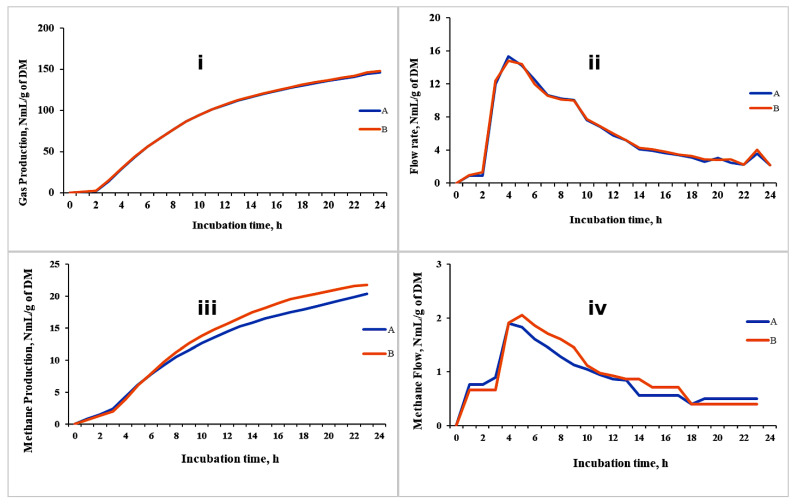
Cumulative GP (**i**), flow kinetics of GP (**ii**), cumulative CH_4_ (**iii**) and flow kinetics of CH_4_ (**iv**) production from a TMR for lactating cows using the GES (unpublished data). A and B are replicates of the same TMR treatment.

The results of the demonstrative trial are presented in Figure 5. The replicates for cumulative gas production and gas flow rates showed high consistency. However, some variation was observed in the replicate values for cumulative methane production and its kinetics, though this variation was negligible. We believe this variation is likely due to the extended travel time required for methane to move from the fermentation unit (Unit A) to the methane measurement cell (Unit C1). To address this, it is recommended to increase the volume of the buffered rumen medium to ensure sufficient gas production and facilitate a quicker transfer from Unit A to Unit C2.

Recommendation: For improved accuracy in methane measurement, we recommend using a higher volume of incubation media. Specifically, volumes exceeding 250 mL are advisable to enhance the sensitivity and reliability of methane measurement. Additionally, employing a smaller headspace volume is suggested to minimize gas dilution and improve the precision of gas quantification.

#### 3.4.4. Real-Time Monitoring

The GES provides, in real time, the total gas and methane production kinetics that can be monitored at every moment. This information can be expressed across various time intervals, such as 1 min, 15 min, and 1 h. The real-time monitoring allows the incubation time to be easily adjusted. The incubation can be stopped or extended depending on the GP curve shown on the screen, so it guarantees a sufficient time of fermentation of the testing material. In fact, some studies recommend stopping the incubation at the peak of the GP rate, at which the efficiency of microbial production is at a maximum [83], to study the microbial profile and activity and the product of fermentation. The GES has not yet been used for this purpose; however, theoretically, it could be applied, offering significant time and cost savings in analysis compared to other *in vitro* systems that lack continuous gas production monitoring. In Figure 5, an example of cumulated gas and methane production throughout a 24 h incubation period is reported.

#### 3.4.5. Limitations

While *in vitro* methodologies are generally less expensive than *in vivo* experiments, the GES has higher initial installation costs compared to other *in vitro* methods [84]. However, these costs can be offset with consistent use across multiple trials, making it cost-effective over time.

The system’s throughput is limited, as it cannot handle as many simultaneous samples as gravimetric or manual systems. With limited bottle capacity, a maximum of four treatments per run is possible, including blanks, controls, and triplicates for statistical validation.

The Tygon tubes that connect the bottles (Unit A) with the flow cells (Unit C-1) often become blocked with water droplets. Water condensation occurs at the very end of the procedure, after the rumen fluid is injected into the bottles. This may be a major concern because these tubes transport the gas produced in the reactors. If they become blocked with water, the gas accumulating in the headspace of the bottles might not be able to pass through and will not arrive to be measured by the flow cells. Also, this might alter the pressure inside the bottles leading to a possible underestimation of the total GP and an overestimation of CH_4_.

Any standardization of the procedure on how to use the GE for feedstuff *in vitro* GP has not been established until now. As with any new equipment, its implementation requires further examination for adapting the technique to the system and also some repetition to refine the method. Even though a full standardization of the technique is not feasible because of issues mostly related to the animal and the rumen fluid, a basic protocol that covers the procedure of incubation is needed. The harmonization of the whole procedure and detailed information regarding analytical procedures will guarantee repeatability and then facilitate the validation of the GES results and the comparison between results from different *in vitro* experiments. Chemical methodologies should not have a problem regarding repeatability, but again, as we are dealing with biological agents such as rumen fluid, results may differ among authors. This is also why a comparison of GP measurements between different laboratories needs to be performed to achieve reproducible results and then validation.

A comparison with some other techniques that have been extensively studied could be conducted in order to validate the GP results from the GES. As has been discussed before, the results coming from volumetric-based methods tend to be higher than the ones coming from pressure-based methods. If this is the case, the GES may be overestimating the GP.

Therefore, further studies are required on the development of a standardized procedure for feedstuff *in vitro* fermentation and the *in vivo* significance of the results obtained.

## 4. Potential Future Applications

The GES, initially developed to measure biomethane potential, has since found applications in various fields such as wastewater treatment, biodegradability testing, and notably, animal nutrition. A quick Google Scholar search shows that there are over 150 publications referencing the GES overall, while animal nutrition-specific studies—including conference proceedings, theses, and journal articles—number around 30. These figures are approximate and depend on the search terms used (e.g., “Gas Endeavour animal nutrition” versus “Gas Endeavour biogas”).

With further development and research, the GES can be used for a variety of objectives across numerous sectors, especially in the ruminant nutrition field. GES applications go beyond measuring ruminant methane generation [47]. Even though it is clear that the GES could play an important role in the study of rumen modulators for increasing the efficiency of microbial protein synthesis and decreasing methane emissions by ruminants, the GES can be useful to provide better insights into other nutritional parameters. Like any other *in vitro* technique, the GES may be used to predict voluntary intake as studied by Blummel et al. [85]. These authors observed a significant correlation between the *in vitro* fermentative properties of the diet and the *in vivo* dry matter intake in cattle [85]. Also, it could be a useful tool to assess the action of antinutritional factors such as alkaloids, tannins, saponins, phenolics, etc., on rumen fermentation. Some ingredients often contain secondary compounds that affect rumen microbes. They are able to dissolve inside the rumen, so their effect is somehow difficult to assess. The GES could provide better insights into nutrient–antinutrient interactions by measuring the effects of additives that can neutralize the influence of antinutrients on microbial efficiency [86]. It could have the ability to predict the digestibility of feeds because *in vitro* rumen GP can accurately predict the metabolizable energy content of a wide variety of feeds [87]. Moreover, the use of GES could contribute to studying the associative effects when mixing different types of feeds in ruminants’ diets because the use of some feed ingredients may alter the digestibility of the others by stimulating rumen fermentation [88]. A further application could involve evaluating the effect of feed processing, such as dry rolling, steaming, flaking, milling, and others, which may alter the starch digestion by microbial enzymes [88]. The GES can assess the kinetics of different grain varieties as a way to select the ones with a greater enzymatic degradation inside the rumen. In this way, the GES could become a potential and valuable tool that links plant breeding programs with ruminant performance. In general, the GES could contribute to the development of nutritional supplementation strategies using locally available conventional and unconventional feedstuffs in order to achieve maximum microbial efficiency in the rumen.

## 5. Conclusions

Ruminant livestock production significantly contributes to global greenhouse gas emissions, primarily through enteric methane (CH_4_) release. Addressing this issue requires precise, cost-effective, and ethically viable methods to quantify and mitigate methane emissions. While *in vivo* techniques remain the benchmark, their limitations have led to the advancement of *in vitro* systems like the Gas Endeavour System (GES), which provides real-time insights into methane kinetics and fermentation dynamics.

The GES offers significant advantages over other *in vitro* methods, particularly its fully automated operation, continuous monitoring, and elimination of post-incubation gas chromatography. Unlike both traditional and fully automated pressure-based methods, which only provide gas production kinetics, the GES delivers direct methane production kinetics, enhancing its applicability across bioenergy and animal nutrition research. Despite challenges, such as initial costs and protocol standardization, its efficiency and precision make it a promising tool for improving methane mitigation strategies.

In conclusion, the Gas Endeavour System represents a pivotal advancement for *in vitro* rumen fermentation research. By combining automation, precision, and real-time analytics, it holds the potential to advance sustainable livestock practices, refine dietary strategies, and contribute meaningfully to global climate change mitigation efforts. As standardization and validation efforts progress, the GES is poised to become a useful tool in both academic and industrial settings, bridging the gap between laboratory insights and practical agricultural solutions.

## Figures and Tables

**Table 2:** Chemical characteristics (% DM) of diet.

	Diet
Dry matter ^1^ (% DM)	58.00
CP ^3^ (% DM)	14.68
EE ^4^ (% DM)	3.41
CF ^5^ (% DM)	5.54
NDF ^6^ (% DM)	41.44
ADF ^7^ (% DM)	21.12
Gross ADL ^8^ (% DM)	4.11
AIA ^9^ (% DM)	0.55
Net ADL (% DM)	3.95
Ash (% DM)	7.52

^1^ DM: dry matter, ^3^ CP: crude protein, ^4^ EE: ether extract, ^5^ CF: crude fibre, ^6^ NDF: neutral detergent fibre determined with heat-stable α-amylase and without sulfite (cellulose, hemicellulose, lignin), ^7^ ADF: acid detergent fibre, ^8^ ADL: acid detergent lignin, and ^9^ AIA: acid insoluble ash.

## Data Availability

Not applicable.
